# Insulin Receptors and Intracellular Ca ^2+^ Form a Double-Negative Regulatory Feedback Loop Controlling Insulin Sensitivity

**DOI:** 10.12688/f1000research.24558.2

**Published:** 2021-01-13

**Authors:** Igor Pomytkin, Vsevolod Pinelis

**Affiliations:** 1 Department of Advanced Cell Technologies, Sechenov First Moscow State Medical University (Sechenov University), Moscow, Moscow, 119991, Russian Federation; 2 National Medical Research Center for Children’s Health, Russian Ministry of Health, Moscow, 119991, Russian Federation

**Keywords:** Insulin, insulin receptor, glutamate, NMDA receptor, Ca2+, double-negative feedback loop, mitochondria, ATP

## Abstract

Since the discovery of insulin and insulin receptors (IR) in the brain in 1978, numerous studies have revealed a fundamental role of IR in the central nervous system and its implication in regulating synaptic plasticity, long-term potentiation and depression, neuroprotection, learning and memory, and energy balance. Central insulin resistance has been found in diverse brain disorders including Alzheimer’s disease (AD). Impaired insulin signaling in AD is evident in the activation states of IR and downstream signaling molecules. This is mediated by Aβ oligomer-evoked Ca ^2+^ influx by activating N-methyl-D-aspartate receptors (NMDARs) with Aβ oligomers directly, or indirectly through Aβ-induced release of glutamate, an endogenous NMDAR ligand. In the present opinion article, we highlight evidence that IR activity and free intracellular Ca ^2+^ concentration [Ca ^2+^] *_i_* form a double-negative regulatory feedback loop controlling insulin sensitivity, in which mitochondria play a key role, being involved in adenosine triphosphate (ATP) synthesis and IR activation. We found recently that the glutamate-evoked rise in [Ca ^2+^] *_i_* inhibits activation of IR and, vice versa, insulin-induced activation of IR inhibits the glutamate-evoked rise in [Ca ^2+^] *_i_*. In theory, such a double-negative regulatory feedback loop predicts that any condition leading to an increase of [Ca ^2+^] _i_ may trigger central insulin resistance and explains why central insulin resistance is implicated in the pathogenesis of AD, with which glutamate excitotoxicity is a comorbid condition. This model also predicts that any intervention aiming to maintain low [Ca ^2+^] _i_ may be useful for treating central insulin resistance.

## Introduction

Since the discovery of insulin ^1^ and insulin receptors (IR) ^2^ in the brain in 1978, numerous studies have revealed a fundamental role of IR in the central nervous system (CNS). IR-mediated signaling is implicated in the regulation of diverse functions in the CNS, including synaptic plasticity, long-term potentiation and depression, neuroprotection, learning and memory, and energy balance ^3^. Central insulin resistance has been found in neurodegenerative diseases such as Alzheimer’s disease (AD) ^4, 5^ and Parkinson’s disease (PD) ^6^, stroke, and traumatic brain injury (TBI) ^7^. Impaired insulin signaling in AD is evident in the activation states of IR and downstream signaling molecules ^5^. Compared with control cases, insulin in AD brains induced 24–58% less activation at the level of IR and 90% less activation of insulin receptor substrate 1 (IRS-1) ^5^. It has been presumed ^5^ that the inhibition of IR activation is mediated by Aβ oligomer-triggered Ca ^2+^ influx, in part by activating N-methyl-D-aspartate receptors (NMDARs) ^8^, followed by a rise in Akt1 pS ^473^ ^9^, which can inhibit insulin-induced IR activation through Thr phosphorylation of the IR β subunit ^10^. Aβ oligomers may activate the NMDAR-gated Ca ^2+^ influx directly ^11^ or indirectly through the intermediate release of glutamate, a ligand of NMDAR ^11– 15^. This suggests that the rise in intracellular free Ca ^2+^ concentration ([Ca ^2+^] _*i*_), evoked by either Aβ oligomers or glutamate, leads to dysfunctional activation of IR in AD. In the present opinion article, we highlight evidence that IR and [Ca ^2+^] _*i*_ form a double-negative regulatory feedback loop controlling insulin sensitivity, and mitochondria have a key role in this feedback loop, being involved in adenosine triphosphate (ATP) synthesis and IR activation.

## Glutamate-evoked rise in [Ca ^2+^] _*i*_ causes inhibition of IR activation

**Figure 1.**
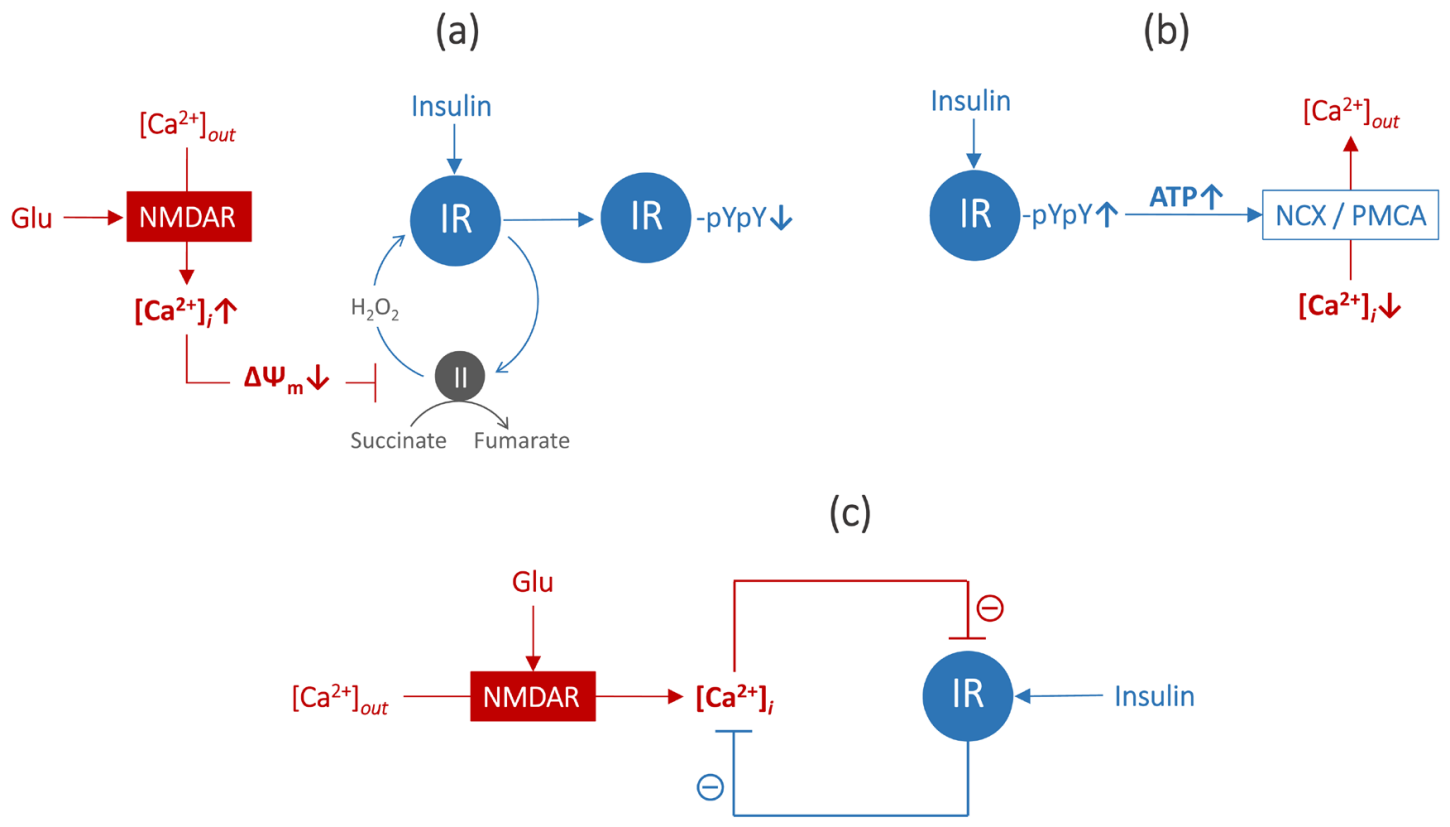
A double-negative feedback loop between insulin receptors (IR) activity and intracellular free Ca ^2+^ concentration [Ca ^2+^] _*i*_. ( **a**) glutamate-evoked increase in [Ca ^2+^] _*i*_, followed by drop in ΔΨ _m_, leads to inhibition of insulin-induced H _2_O _2_ signal at mitochondrial complex II, thereby inhibiting IR activation (IR-pYpY↓) ^20, 21^; ( **b**) insulin-triggered increase in ATP levels enhances ATP-dependent Ca ^2+^ efflux via PMCA and NCX, thereby lowering [Ca ^2+^] _*i*_^19^; ( **c**) glutamate triggers NMDA receptor-gated Ca ^2+^ influx, inhibiting IR activation, and insulin triggers activation of IR, inhibiting [Ca ^2+^] _*i*_ rise. Abbreviations: ΔΨ _m_ , mitochondrial inner membrane potential; H _2_O _2_, hydrogen peroxide; pY, phosphotyrosine; ATP, adenosine triphosphate; PMCA, plasma membrane Ca ^2+^ ATPase; NCX, Na ^+^/Ca ^2+^ exchanger; NMDA, N-methyl-D-aspartate.

Glutamate serves as the major excitatory neurotransmitter in the CNS. Its excessive accumulation in a synaptic cleft can trigger excitotoxicity, a pathologic process leading to neuronal cell death. Glutamate-induced activation of the NMDAR-gated Ca ^2+^ influx is generally considered central to the development of excitotoxicity ^16^. Prolonged glutamate exposure causes a rapid initial increase in the [Ca ^2+^] _*i*_, followed by a larger secondary [Ca ^2+^] _i_ increase concomitant with a decrease in the mitochondrial inner membrane potential (ΔΨ _m_) ^17– 19^. We recently found that on Ca ^2+^-induced mitochondrial depolarization, insulin induced 48% less activation of IR (assessed by pY ^1150/1151^) compared with control ^20^. Earlier, we showed that a decrease in ΔΨ _m_ can abrogate IR activation ^18^, since the ΔΨ _m_-dependent hydrogen peroxide (H _2_O _2_) mitochondrial signal at complex II is critically involved in the activation of IR in neurons ^21– 23^. Thus, the glutamate-evoked increase in [Ca ^2+^] _*i*_, followed by the drop in ΔΨ _m_, leads to the inhibition of insulin-induced activation of IR ( Figure 1a).

## Insulin prevents glutamate-evoked rise in [Ca ^2+^] _*i*_

Normally, the NMDAR-gated Ca ^2+^ influx is counterbalanced with Ca ^2+^ efflux, which is governed by plasma membrane Ca ^2+^ ATPase (PMCA) and the Na ^+^/Ca ^2+^ exchanger (NCX) ^24, 25^. NCX-mediated Ca ^2+^ efflux is also ATP-dependent, since NCX exchanges one Ca ^2+^ for three Na ^+^, and the three Na ^+^ are then pumped out by the Na ^+^/K ^+^ ATPase at the expense of one ATP. In excitotoxicity, prolonged stimulation with glutamate leads to ATP depletion and an abnormal rise in [Ca ^2+^] _i_, since the massive Ca ^2+^ influx is no longer counterbalanced by Ca ^2+^ efflux ^26^. Therefore, maintenance of ATP production is crucial for preventing the rise in [Ca ^2+^] _i_ in excitotoxicity. We found recently that pre-treatment with insulin prevents neurons from glutamate-evoked ATP depletion due to its protective effect on spare respiratory capacity (SRC), a measure that relates to the amount of extra ATP that can be produced via oxidative phosphorylation in case of increased energy demand ^19^. The effect of insulin on SRC relates to its action on mitochondrial metabolism. It has long been known that the tricarboxylic acid cycle is the intracellular site of insulin action and that insulin acutely stimulates succinate oxidation at mitochondrial complex II ^26, 27^. Succinate oxidation at mitochondrial complex II has been identified recently as the main source of SRC ^28^. In line with this, insulin prevented the glutamate-evoked rise in [Ca ^2+^] _*i*_ in our experiments with glutamate excitotoxicity ^19^ ( Figure 1b).

## IR and [Ca ^2+^] _*i*_ form a double-negative feedback loop controlling insulin sensitivity

Collectively, this evidence suggests that a double-negative regulatory feedback loop exists between IR activity and [Ca ^2+^] _*i*_. The glutamate-evoked rise in [Ca ^2+^] _*i*_ inhibits activation of IR and, vice versa, insulin-induced activation of IR inhibits the glutamate-evoked rise in [Ca ^2+^] _*i*_ ( Figure 1c).

This double-negative feedback loop model predicts that any condition leading to an increase in [Ca ^2+^] _i_ may trigger insulin resistance. It appears to explain why central insulin resistance is implicated in the pathogenesis of disorders such as AD ^4, 5^, PD ^6^, stroke, and TBI ^7^, with which glutamate excitotoxicity is a comorbid condition ^29^. The model also predicts that any intervention aiming to prevent Ca ^2+^ influx of or enhance efflux of Ca ^2+^ from neurons, thereby maintaining low [Ca ^2+^] _i_, may be useful for treating central insulin resistance. Given that Ca ^2+^ efflux is ATP-dependent, any intervention directed to enhance ATP production in neurons may be especially useful to improve insulin sensitivity in the brain. 

## Data availability

No data are associated with this article.
