# Supplementary material for: Insulin Receptors and Intracellular Ca 2+ Form a Double-Negative Regulatory Feedback Loop Controlling Insulin Sensitivity
Source: F1000Res. 2021 Jan 13;9:598. Originally published 2020 Jun 12. [Version 2] doi: 10.12688/f1000research.24558.2 (PMC7845146; doi:10.12688/f1000research.24558.2)
Supplement: Peer review history

# Peer review: Reviewer response for version 2

**DOI:** 10.5256/f1000research.52891.r77179

**Published:** 2021-01-29

**Authors:** Kevin N. Hascup

**Affiliations:** 1 Department of Neurology, Center for Alzheimer's Research and trEatment (CARE), Springfield, IL, USA

Although the authors have adequately presented point-by-point responses to my initial concerns, they have taken a minimalistic approach to incorporating some of these concepts into the actual manuscript.  It would have been interesting to read the authors ideas on how this double feedback loop is challenged in different disease states considering this forms the premise of the introduction and the reasoning behind the opinion piece. Regardless, in situations of extreme deviations from homeostatic calcium concentrations (stroke and traumatic brain injury) concerns about cerebral insulin resistance is minimal compared to the rapid loss of nervous tissue. This double feedback loop postulate may be better suited for slower progressing neurodegenerative disorders.

Is the topic of the opinion article discussed accurately in the context of the current literature?

Partly

Are arguments sufficiently supported by evidence from the published literature?

No

Are all factual statements correct and adequately supported by citations?

Yes

Are the conclusions drawn balanced and justified on the basis of the presented arguments?

Partly

Reviewer Expertise:

glutamate signaling, Alzheimer's disease, Parkinson's disease, insulin signaling, gerontology

I confirm that I have read this submission and believe that I have an appropriate level of expertise to confirm that it is of an acceptable scientific standard.

# Peer review: Reviewer response for version 2

**DOI:** 10.5256/f1000research.52891.r77181

**Published:** 2021-01-27

**Authors:** Venkatesh V. Kareenhalli

**Affiliations:** 1 Indian Institute of Technology Bombay, Mumbai, Maharashtra, India

The manuscript can now be accepted as all concerns have been addressed.

Is the topic of the opinion article discussed accurately in the context of the current literature?

Yes

Are arguments sufficiently supported by evidence from the published literature?

Yes

Are all factual statements correct and adequately supported by citations?

Yes

Are the conclusions drawn balanced and justified on the basis of the presented arguments?

No

Reviewer Expertise:

Systems biology, Liver metabolism, modeling signaling pathways

I confirm that I have read this submission and believe that I have an appropriate level of expertise to confirm that it is of an acceptable scientific standard.

# Peer review: Reviewer response for version 1

**DOI:** 10.5256/f1000research.27092.r72629

**Published:** 2020-11-02

**Authors:** Venkatesh V. Kareenhalli

**Affiliations:** 1 Indian Institute of Technology Bombay, Mumbai, Maharashtra, India

The authors propose an existence of double negative feedback loop which may result in bistable response. It is clear that Ca signaling and insulin signaling play a role in the CNS and the related pathogenesis of neurogenerative diseases. The mitochondrial function and thereby ATP synthesis also plays a role in the stated phenotype. The proposed dual negative feedback on each other can toggle between two states, but it may not be bistable. Bistable is with respective to an input variation. It is unclear what is the input that the hypothesis is being discussed. At a given input, the existence of both steady state, with different history, yields bistability. A figure that demonstrates all the molecular components and action including mitochondrial state and metabolites is more helpful than what is illustrated.

Is the topic of the opinion article discussed accurately in the context of the current literature?

Yes

Are arguments sufficiently supported by evidence from the published literature?

Yes

Are all factual statements correct and adequately supported by citations?

Yes

Are the conclusions drawn balanced and justified on the basis of the presented arguments?

No

Reviewer Expertise:

Systems biology, Liver metabolism, modeling signaling pathways

I confirm that I have read this submission and believe that I have an appropriate level of expertise to confirm that it is of an acceptable scientific standard, however I have significant reservations, as outlined above.

# Response

**Published:** 2021-01-08

**Authors:** Igor Pomytkin

**Affiliations:** 1Department of Advanced Cell Technologies, Sechenov First Moscow State Medical University (Sechenov University), 119991, Moscow, Russia., Russian Federation

The manuscript has been revised in accordance with notes that (a) bistability is not a necessary consecuence of a double negative regulatory feedback loop and (b) figure 1 will be more useful when signal transduction pathways will be shown. Sentences related to "bistability" have been removed from the abstract, main text, and reference list. Figure 1 has been revised and includes now pathways described in the text of the first version.

# Peer review: Reviewer response for version 1

**DOI:** 10.5256/f1000research.27092.r67002

**Published:** 2020-08-03

**Authors:** Zhen Deng

**Affiliations:** 1 Department of Neurology, Nanfang Hospital, Southern Medical University, Guangzhou, China

This model predicts that any disease that causes elevated [Ca ^2+^] _i_ may trigger central insulin resistance, and explains why central insulin resistance is related to the pathogenesis of AD, and glutamate excitotoxicity is a comorbidity. The model also predicts that any intervention aimed at maintaining low [Ca ^2+^] _i_ can be used to treat central insulin resistance.

 It is an interesting theory between brain insulin resistance and AD. Although not much experimental data support the theory directly, it is worth following.

Is the topic of the opinion article discussed accurately in the context of the current literature?

Yes

Are arguments sufficiently supported by evidence from the published literature?

Yes

Are all factual statements correct and adequately supported by citations?

Yes

Are the conclusions drawn balanced and justified on the basis of the presented arguments?

Yes

Reviewer Expertise:

cerebral vascular disease

I confirm that I have read this submission and believe that I have an appropriate level of expertise to confirm that it is of an acceptable scientific standard.

# Peer review: Reviewer response for version 1

**DOI:** 10.5256/f1000research.27092.r64837

**Published:** 2020-07-01

**Authors:** Kevin N. Hascup

**Affiliations:** 1 Department of Neurology, Center for Alzheimer's Research and trEatment (CARE), Springfield, IL, USA

The authors put forth an opinion article in regard to a double negative feedback loop that controls cerebral insulin receptor signaling during times of either high glutamate or insulin levels. They briefly go onto hypothesize that any disease state resulting in elevated intracellular calcium will result in reduced cerebral insulin resistance, particularly when excitotoxicity is a comorbid factor. The authors attempt to simplify an extremely complicated phenomenon. However, this oversimplification neglects to take into account several factors that need to be addressed.

 Reduced insulin signaling as a result for glutamate induced calcium influx has been discussed in other literature (see Zhao *et al.*, 2008 ^1^). This work should be taken into consideration when authors discuss their model.

 Although insulin receptors are ubiquitously expressed in different cell types of the CNS, these cell types rely on different mechanisms for ATP production. Glial cells predominantly use glycolytic pathways in the cytoplasm whereas neurons rely on oxidative phosphorylation in the mitochondria. (For review see Pellerin and Magistretti, 2012 ^2^). Accordingly, the cellular localization of ATP production should be taken into consideration. Furthermore, do the authors believe different cell type specific mechanisms exist that their model should take into consideration?

 In several places, the authors discuss the role of amyloid on activation of NMDA receptors and the subsequent intracellular Calcium increase. However, the authors have not taken into account how amyloid binding can also elicit glutamate release from either α7nAChRs (see Hascup and Hascup, 2016 ^3^ and Mura *et al.*, 2012 ^4^) or mGLUR5 receptors (Renner *et al.*, 2010 ^5^). In the latter case, several laboratories have shown mGLUR5 acts as a scaffolding complex for amyloid accumulation causing receptor clustering at the membrane surface and results in elevated intracellular calcium levels. 

 Additionally, the manuscript could be improved if a discussion on how their model might vary across different CNS disorders such as AD, Parkinson’s TBI, etc. in relation to healthy functional activity.

 The authors neglect to take into account factors that also play a role in modulating insulin signaling. These include:

- Inflammation. This is prominent in numerous disease states and can negatively impact insulin signaling, while exacerbating mechanisms associated with multiple neurodegenerative disorders.
- Inhibitory feedback regulation. Insulin signaling is tightly controlled to prevent perturbations in metabolism as well as control the specificity of the signal on multiple downstream effectors. Several phosphatases are responsible for this, not just at the receptor, but also on effector enzymes.The current model does not take into consideration this tightly controlled feedback loop.
- Receptor internalization. Upon insulin binding, the insulin receptor becomes internalized as another means to control the strength and duration of the signal. This internalization is more prominent in hyperinsulinemia and may account for the resulting insulin resistance. How would this model change during stages of insulin resistance, which are hypothesized to initiate the cognitive decline observed in AD?
- Peripheral insulin signaling.Insulin produced in the pancreas is able to enter the CNS.How does the proposed model take into consideration fluctuations during normal periods of food consumption?

Are the authors proposing a similar mechanism for the structurally analogous insulin-like growth factor-1?

Minor concerns:

- The glutamate pathway in Figure 1 should have a different color scheme to make it easier to differentiate from Calcium concentration.
- Figure 1B is slightly confusing. The red line makes it seem that low levels of glutamate give rise to high levels of Calcium instead of showing just the rise in calcium. A way to incorporate glutamate activation of NMDA receptors in Fig 1B & C might help to conceptualize the model. 
- The abstract is lengthy in relation to the article. I would suggest this is shortened and made more succinct.

Is the topic of the opinion article discussed accurately in the context of the current literature?

Partly

Are arguments sufficiently supported by evidence from the published literature?

No

Are all factual statements correct and adequately supported by citations?

Yes

Are the conclusions drawn balanced and justified on the basis of the presented arguments?

Partly

Reviewer Expertise:

glutamate signaling, Alzheimer's disease, Parkinson's disease, insulin signaling, gerontology

I confirm that I have read this submission and believe that I have an appropriate level of expertise to state that I do not consider it to be of an acceptable scientific standard, for reasons outlined above.

## References

[1] : Amyloid beta oligomers induce impairment of neuronal insulin receptors. *FASEB J*.2008;22(1) : 10.1096/fj.06-7703com 246-60 10.1096/fj.06-7703com 17720802

[2] : Sweet sixteen for ANLS. *J Cereb Blood Flow Metab*.2012;32(7) : 10.1038/jcbfm.2011.149 1152-66 10.1038/jcbfm.2011.149 22027938PMC3390819

[3] : Soluble Amyloid-β42 Stimulates Glutamate Release through Activation of the α7 Nicotinic Acetylcholine Receptor. *Journal of Alzheimer's Disease*.2016;53(1) : 10.3233/JAD-160041 337-347 10.3233/JAD-160041 27163813

[4] : Dual effect of beta-amyloid on α7 and α4β2 nicotinic receptors controlling the release of glutamate, aspartate and GABA in rat hippocampus. *PLoS One*.2012;7(1) : 10.1371/journal.pone.0029661 e29661 10.1371/journal.pone.0029661 22253754PMC3256170

[5] : Deleterious effects of amyloid beta oligomers acting as an extracellular scaffold for mGluR5. *Neuron*.2010;66(5) : 10.1016/j.neuron.2010.04.029 739-54 10.1016/j.neuron.2010.04.029 20547131PMC3111138

# Response

**Published:** 2020-07-10

**Authors:** Igor Pomytkin

**Affiliations:** 1Department of Advanced Cell Technologies, Sechenov First Moscow State Medical University (Sechenov University), 119991, Moscow, Russia., Russian Federation

Our opinion article relates only to relationship between intracellular Ca ^2+^ concentrations [Ca ^2+^]i and insulin receptor activation state, and not between [Ca ^2+^]i and insulin signaling system as a whole. Insulin resistance is the term that currently applied to any of the biological actions of insulin and, therefore, is too broad to be discussed in terms of models that may predict the system behavior. The proposed model in the opinion article is not oversimplified, but takes into consideration only link between [Ca ^2+^]i and the stage of activation of insulin receptor kinase (i.e Tyr1150/1151 phosphorylation), the earliest step in insulin action that precedes all other signaling events and effects of insulin.

In our opinion article we selected only two measurable parameters, namely [Ca ^2+^]i and insulin receptor activation state defined as Tyr1150/1151 phosphorylation, but not downstream molecules of IR signaling pathway such as IRS-1 or others. Therefore, our opinion relates only to the activation of insulin receptor, and not to downstream molecules or events. This approach relates directly to insulin sensitivity, since the activation of the receptor with insulin is the only stage at which insulin sensitivity can be measured directly.

The opinion about existence of regulatory double-negative feedback loop between [Ca ^2+^]i and insulin receptor activation state is based on our experimental results, obtained at the same experimental conditions in two studies that have already been published [references 19 and 20].

Results of Zhao et al. do not contradict our opinion about relationship between Ca ^2+^ and insulin receptor activation state. Moreover, results of Zhao et al. support our opinion. Zhao et al. have found that glutamate stimulation reduced the insulin-stimulated tyrosine phosphorylation of the receptor  beta-subunit  at Tyr1150/1151 and this glutamate effect was completely inhibited by the cell-permeable Ca ^2+^ chelator BAPTA-AM. Although authors did not measure  intracellular Ca ^2+^ concentrations, they concluded that IR inhibition is dependent on elevated intracellular Ca ^2+^, given a well-known link between glutamate and  Ca ^2+^. It is the same conclusion that we made. The difference is only that our conclusion is made on our direct measurements of intracellular Ca ^2+^.

In our studies [references 19 and 20] that underlie the our opinion we used glia-free cortical neurons and directly measured ATP levels.

Our opinion relates to link between [Ca ^2+^]i and insulin receptor activation state, and amyloid-NMDA relationship are out of scope of our opinion.

Our opinion is limited to only insulin receptor activation, but not to more broad “insulin signaling”.  According to current knowledge, inflammation affects insulin signaling, but downstream of insulin  receptor at IRS-1 level. This is out of scope of our opinion.

Our opinion does not relate to any signaling events downstream of insulin receptor, such phosphatase action and receptor internalization. 

The insulin regulated internalization of insulin receptors has been shown to require autophosphorylation of all three regulatory tyrosines 1146, 1150, and 1151. Carpentier JL et al. Two steps of insulin receptor internalization depend on different domains of the beta-subunit. J Cell Biol. 1993 Sep;122(6):1243-52. doi: 10.1083/jcb.122.6.1243. Thus, the internalization occurs after the activation of IR and, therefore, is out of the scope of our opinion.

According to the model, insulin inhibits rise of [Ca ^2+^]i independently of the insulin source. Therefore, during periods of insulin transport to the brain, the rise of [Ca ^2+^]i , e.g. glutamate-evoked, would be diminished, independently on whether it comes from pancreas or administered intranasally.

We did not propose the same mechanism for IGF-1 in our opinion aticle, since we have no supportive evidence. However, it is likely that there is a link between [Ca ^2+^]i and activation state of IGFR1.

In a conclusion, the model proposed in the opinion article relates only to relationship beteen two measurable parameters, namely intracellular Ca2+ concentrations and insulin receptor activation state. Therefore, all other downstream elements of insulin signaling system and factors affecting the downstream elements are out of scope of this opinion. The opinion is completely based on our experimental results previously published, references 19 and 20 of the opinion article.
